# Formulation and Evaluation of Liposome‐Encapsulated *Lactiplantibacillus plantarum* Postbiotics on Gluten‐Induced Celiac Disease in Caco‐2 Cells via Reduction of Zonulin Secretion

**DOI:** 10.1049/nbt2/8828841

**Published:** 2026-10-06

**Authors:** Mojdeh Safari, Mahdi Adabi, Naheed Mojgani, Fatemeh Pirnajafi, Masoomeh Amini, Milad Sadeghzadeh, Mohaddeseh Khadem, Bijan Shahbazkhani

**Affiliations:** ^1^ Finetech in Medicine Research Center School of Medicine Iran University of Medical Sciences Tehran Iran iums.ac.ir; ^2^ Department of Medical Nanotechnology School of Advanced Technologies in Medicine Tehran University of Medical Sciences Tehran Iran tums.ac.ir; ^3^ Biotechnology Department Razi Vaccine and Serum Research Institute Agriculture Research, Education and Extension Organization Karaj Iran rvsri.ir; ^4^ Department of Pathobiology School of Public Health Tehran University of Medical Sciences Tehran Iran tums.ac.ir; ^5^ Department of Immunology School of Public Health Tehran University of Medical Sciences Tehran Iran tums.ac.ir; ^6^ Department of Medical Nanotechnology TeMS.C. Islamic Azad University Tehran Iran ctb.iau.ir; ^7^ Digestive Diseases Research Center Shariati Hospital Tehran University of Medical Sciences Tehran Iran tums.ac.ir

**Keywords:** Caco-2 cells, celiac disease, *Lactiplantibacillus plantarum*, liposome, nanostructure, postbiotic, probiotic

## Abstract

Celiac disease (CD) is a severe autoimmune disorder induced by gluten exposure and a complex interplay of environmental contributors and genetics. While a gluten‐free diet (GFD) is the primary approach for managing CD, it is relatively costly, inconvenient, and challenging to follow. Recently, emerging studies have focused on pro‐, pre‐, and postbiotics, which have been demonstrated to break down gluten and provide a protective effect against the damage exerted by gluten. Thus, this study aimed to utilize postbiotic fractions obtained from the probiotic bacterium *Lactiplantibacillus plantarum* in the management of CD. The efficacy of the resulting postbiotic in both free and liposomal forms has been studied on the Caco‐2 cell line as a model of CD. The particle size and Z potential of liposome‐encapsulated postbiotic were assessed at about 289 ± 9.6 nm and −70.1 ± 3.8 mV, respectively. An encapsulation efficiency (EE, %) of 81.83 ± 3.2% and a sustained release profile were noted for the liposome‐encapsulated postbiotic. The MTT assay was employed to evaluate the cytotoxic effects of free and liposomal postbiotic on Caco‐2 cells. The free and liposomal postbiotic demonstrated less toxicity than gliadin toward the investigated cell line. Importantly, cotreatment of free and liposomal postbiotics with gliadin remarkably reduced the toxic effects of gliadin. The findings of this research revealed that the evaluated postbiotic from *L. plantarum*, particularly in its liposomal form, can significantly reduce zonulin secretion and have a protective effect against gluten‐induced damage.

## 1. Introduction

Celiac disease (CD), or gluten‐sensitive enteropathy (GSE), is a chronic inflammatory disorder that impacts the small intestine due to an autoimmune response triggered by gluten consumption, a protein found in wheat, barley, and several other cereal grains [1, 2]. Its global prevalence is influenced by environmental factors (gluten), genetic factors (HLA‐DQ2/DQ‐positive and non‐HLA genes), viral infections, and gut microbiota dysbiosis [3, 4]. Gliadin peptides are resistant to digestive enzymes and stomach acid, thereby remaining intact in the intestinal lumen following gluten ingestion [5]. CD is also characterized by increased paracellular permeability and a disruption in the integrity of the intestinal barrier. The integrity of the intestinal barrier depends on a complex of proteins forming various intercellular junctions, such as adherens junctions and tight junctions (TJs) [6]. As a regulatory protein, zonulin shows the capability to reversibly adjust intercellular TJs [7]. Zonulin stimulates the epidermal growth factor receptor (EGFR) via the proteinase‐activated receptor 2 (PAR 2), which leads to the phosphorylation of TJ proteins and rearrangement of actin filaments [8, 9], subsequently causing the inhibition of TJ proteins. Moreover, gliadin promotes zonulin signaling in CD patients, resulting in elevated intestinal permeability [10]. A strict gluten‐free diet (GFD) remains the gold‐standard treatment for CD, which often leads to intestinal dysbiosis [11, 12]. Despite the considerable advancements achieved in treating CD with zonulin inhibitors, genetically altered gluten, tissue transglutaminase inhibitors, and therapeutic vaccines, there is still an urgent need for innovative therapeutic strategies [13]. Recently, probiotic bacteria have been extensively studied in several gastrointestinal diseases. In vitro and in vivo studies demonstrate their capacity to maintain the intestinal barrier, improving its integrity in both conditions [14]. Probiotics are live microorganisms that confer several health benefits when administered in sufficient quantities [15]. The imbalance in the gut microbiome in patients with CD has resulted in the exploration of probiotics as a potential alternative [16]. Probiotics have a significant effect on reducing the secretion of zonulin [17]. Various probiotic strains can mitigate gliadin’s harmful effects, and *Lactiplantibacillus plantarum*, a key probiotic in the digestive system, could provide therapeutic benefits for CD [18, 19]. Although probiotics are known to positively impact the microbial balance of the gut and offer several beneficial effects for the host, concerns have arisen regarding the use of live microorganisms for those with compromised immune systems [20]. Moreover, these live microorganisms encounter obstacles to survival and possess a limited shelf life [21]. Emerging studies highlight the beneficial health impacts of inactivated probiotic bacterial cells and their metabolites, commonly known as postbiotics. According to the International Scientific Association for Probiotics and Prebiotics (ISAPP), “a postbiotic is defined as a preparation of nonliving microorganisms and/or their cellular components that provides a health advantage to the host” [22]. Due to their desirable characteristics, including established chemical structures, long storage stability, nontoxicity, and resistance to intestinal enzymes, they represent a safer therapeutic alternative to viable probiotics for clinical applications. The main mechanism of postbiotics involves regulating immune responses, providing protection against the toxic effects of gliadin, modulating the gut microbiota composition, and reducing intestinal permeability [14, 23]. To date, limited knowledge exists regarding the impact of postbiotics on CD, with only a few studies conducted on this topic. Sarno et al. [24] have shown that the *Lactobacillus paracasei* postbiotics effectively decrease the entry of gliadin peptides into intestinal epithelial cells. Despite the promising effects of free postbiotics, their clinical application is often limited by rapid degradation, poor bioavailability, and a short residence time in the gastrointestinal tract [25]. The encapsulation of pro‐, pre‐, or postbiotics has been considered as a novel approach for successful delivery towards their targeted release in the host intestine. One of the most attractive advantages of encapsulation is associated with enhanced stability, improved bioavailability, and controlled release of encapsulated bioactives at the desired physiological location [26]. Among drug delivery systems, liposomes are the most commonly applied carriers for various substances, including nutraceuticals, drugs, enzymes, and beneficial microorganisms. Hence, encapsulation of probiotics and postbiotics in liposomal structures is of great interest [27, 28]. Liposomes are nanoscale (50–500 nm) spherical vesicles characterized by one or more bilayers of phospholipids [29, 30]. Liposomes are capable of enclosing components with various polarities, generating a natural barrier that safeguards bioactives from external stimuli [31, 32]. Thus, we hypothesized that liposomal encapsulation would not only protect the postbiotic bioactive components from enzymatic and pH‐induced degradation but also provide a sustained release profile, thereby enhancing their protective efficacy against gliadin‐induced intestinal damage compared to the free form; accordingly, in this study, postbiotics obtained from *L. plantarum* were encapsulated in a liposomal structure. The liposomal postbiotics were then characterized for their particle size, zeta potential, encapsulation efficiency (EE), and drug release. The cytotoxicity of free postbiotics and liposomal postbiotics was evaluated on the Caco‐2 cell line as an in vitro model of CD. The zonulin levels were also assessed after treatments with free and liposomal postbiotics.

## 2. Experimental Section

### 2.1. Raw Materials and Reagents

All chemicals were of analytical grade and used as received without further purification, unless otherwise stated. Fetal bovine serum (FBS) (GIBCO/BRL Invitrogen, Carlsbad, California), trypsin (Biosera, England), penicillin‐streptomycin (Biosera, England), DMEM‐F12 (GIBCO/BRL Invitrogen, Carlsbad, California), methylthiazolyl diphenyltetrazolium bromide (98%, Sigma–Aldrich, USA), sodium hydroxide (NaOH, Sigma‐Aldrich, USA), acetone (Sigma‐Aldrich, USA), soybean lecithin (Sigma‐Aldrich, USA) and glycerol (Sigma‐Aldrich, USA) were purchased from the indicated suppliers. The bacterial culture media were obtained from Ibresco, Iran.

### 2.2. Probiotic Bacterial Strains

*L. plantarum* (LRH‐B2) used in this study was obtained from a local probiotic manufacturer (Biorun Co., Karaj, Iran). The strain was cultivated in de Man‐Rogosa and Sharpe (MRS) broth (pH 6.8) at 37 °C for 24 h under anaerobic conditions. The stock cultures were maintained in a 20% (v/v) glycerol medium at −20°C.

### 2.3. Preparation of Postbiotic

The postbiotics from *L. plantarum* were prepared according to the method described earlier [33]. Briefly, freshly grown cultures of the mentioned bacterial strain were centrifuged (8000 × *g* for 15 min at 4°C), and the resulting pellets were diluted to 10^9^ colony‐forming units (CFUs) per mL using a sterile sodium chloride solution (NaCl, 0.85% w/v). The prepared bacterial cultures were later treated with lysozyme (1 mg/mL) for 30 min at 37°C, followed by ultrasonic disruption. The suspension was sonicated in an ice bath at 5‐min intervals, and the cell debris was eliminated through centrifugation (8000 × *g* for 15 min at 4°C). The obtained supernatant was subsequently filter sterilized using 0.22 µm Millipore membranes (Sartorius Minisart, Germany) and freeze‐dried as described by Moradi et al. [34]. Following the quality control assessment of the lyophilized powder (FD‐P), the postbiotic fractions free of viable cells were stored at −20°C for future use.

### 2.4. Preparation of Liposome‐Encapsulated Postbiotic

In this research, the liposome‐encapsulated postbiotic was prepared using the Mozafari technique [35, 36]. One of the key benefits of this technique is the preparation of liposomes without the need for detergents or solvents. In addition, this technique does not require sonication or homogenization methods to reduce liposomal particle sizes, as these methods can lead to the destruction of liposome structure. The liposomes were composed of soy lecithin and glycerol. Firstly, soy lecithin and glycerol were mixed in a mass ratio of 1:3 (lecithin:glycerol) with magnetic stirring at 300 rpm for 24 h, until a creamy and uniform mixture was obtained. Then, the solution containing postbiotic and water (at a mass ratio of 50:1) was added dropwise to the initial liposome solution, under continuous stirring (300 rpm) for 2 h. Finally, the liposome‐encapsulated postbiotic was stored at 3–7 °C.

### 2.5. Characterization of Liposome‐Encapsulated Postbiotic

The hydrodynamic diameter of the liposome‐encapsulated postbiotic was determined using dynamic light scattering (DLS), and zeta potential measurement was performed using a Zetasizer (Malvern Instruments, UK). The size and morphological characteristics of the synthesized liposome were analyzed using transmission electron microscopy (TEM).

### 2.6. Standard Curve of Postbiotic

Various concentrations of *L. plantarum* postbiotic (2.5, 2.3, 2.1, 1.9, 1.7, and 1.5 mg/mL) were prepared, and the absorbance was assessed using the spectrophotometer technique at a wavelength of 276.5 nm. The standard curve of postbiotic was generated by plotting absorbance values on the *Y*‐axis against concentrations on the *X*‐axis.

### 2.7. Determination of Drug Loading and Entrapment Efficacy

The liposome‐encapsulated postbiotic suspension was centrifuged at 10,000 rpm for 60 min. The absorbance of the supernatant containing the free postbiotic was measured using UV spectrophotometer at the wavelength of 276.5 nm [37, 38]. The DL% and EE% of the liposome‐encapsulated postbiotic were calculated using the following equations:

Drug loading (wt%)=initial amount of postbiotic mg−amount of free postbiotic mginitial amount of postbiotic mg ×100,

Encapsulation efficiency (wt%)=amount of postbiotic in liposome amount of recovered liposomes×100.

### 2.8. In Vitro Release Study

The release study of the liposome‐encapsulated postbiotic was performed in triplicate using the dialysis membrane method as a standard technique for evaluating the release profiles of encapsulated bioactive compounds [39]. In this procedure, the prepared sample was poured into a dialysis bag with a molecular weight cut‐off (MWCO) of 12 kDa and was immersed in phosphate‐buffered saline (PBS) at pH 1.2, 6.8, and 7.4 in a shaking incubator for 72 h at 37°C and 100 rpm (Labtech, S. Korea). At specified time intervals (0, 1, 2, 4, 6, 12, 24, 48, and 72 h), samples were taken from the release media and replaced with an equal volume of fresh medium. The collected samples were analyzed using UV‐visible spectroscopy at 276.5 nm, and the percentage of postbiotic release was calculated using the postbiotic standard curve in PBS.

## 3. Cell Culture Conditions

Caco‐2 cells, a human colon adenocarcinoma line, were obtained from the Pasteur Institute of Iran. Caco‐2 cells were grown as monolayers in DMEM‐F12 medium containing 10% FBS, 2 mM glutamine, and antibiotics including 100 IU/mL penicillin and 100 μg/mL streptomycin and incubated at 37°C in a humidified 5% CO_2_ atmosphere. The Caco‐2 cells were harvested upon reaching 80% confluence by trypsinization for subsequent steps of this study.

### 3.1. In Vitro Cytotoxicity Evaluation of Liposome‐Encapsulated Postbiotic

The cytotoxic effects of the optimized liposome were assessed in Caco‐2 cell line via the MTT assay. This method quantifies mitochondrial activity through the reduction of 3‐(4,5‐dimethylthiazol‐2‐yl)‐2,5‐diphenyltetrazolium bromide (MTT) into a purple formazan product, primarily due to the function of the mitochondrial enzymes succinate dehydrogenase and cytochrome oxidase within living cells. In brief, Caco‐2 cells were seeded at a density of 5000 cells/well in 96‐well plates and allowed to adhere during a 24 h incubation at 37°C. Subsequently, the old media was discarded, and 100 μL of various concentrations (1, 2, and 4 mg/mL) of free postbiotic, gliadin peptide, and liposomal formulation in the DMEM‐F12 medium was introduced to Caco‐2 cells, separately. The postbiotic concentrations (1, 2, and 4 mg/mL) were selected based on preliminary dose–response experiments. At specified time intervals (24 and 48 h), 20 μL of MTT (5 mg/mL in PBS) was introduced to each well, followed by 4 h of incubation at 37°C under 5% CO_2_–humidified conditions. Afterward, the supernatant containing MTT was discarded, and formazan precipitates were dissolved in 100 μL dimethyl sulfoxide (DMSO). Eventually, the absorbance was quantified at 570 nm using a microplate reader (Biotek, United States). Three independent replicates were conducted, and the percentage of cytotoxicity was determined according to the following formula: 

Cell viability %=Optical density in sample wellOptical density in control well.

## 4. Zonulin Determination

The zonulin levels in the cell culture media were quantified at various time points (3, 6, and 12 h) following treatments with postbiotic and liposome‐encapsulated postbiotic, using the zonulin enzyme–linked immunosorbent assay (human zonulin ELISA) kit (MyBiosource, USA) according to the manufacturer’s instructions.

## 5. Statistical Analysis

All experimental procedures were performed in triplicate, and data are presented as mean ± standard error of the mean (SEM). Data were analyzed using GraphPad Prism version 10, considering *p*‐value of  < 0.05 as statistically significant. The Kruskal–Wallis *H* test was used to analyze the results of the MTT assay, while two‐way ANOVA was employed for the analysis of zonulin release as measured by ELISA.

## 6. Results and Discussions

### 6.1. Physicochemical Characterization of Liposome‐Encapsulated Postbiotic

The biological efficacy of liposomes is significantly influenced by their physicochemical characteristics, such as hydrodynamic diameter, polydispersity index (PDI), and zeta potential. DLS results indicated that the liposome‐encapsulated postbiotic exhibited a uniform size profile, with almost all nanoliposomes distributed in less than 1000 nm. The mean hydrodynamic diameter and PDI were 289 ± 9.6 and 0.13 ± 0.02 nm, respectively. In fact, PDI is an indicator of the particle size homogeneity in the sample; thus, its value can represent the uniform size distribution of the synthesized liposomes. Generally, the liposomes prepared by the Mozafari technique possess acceptable PDI and relatively monodisperse particles. In this research, the zeta potential of the liposome‐encapsulated postbiotic was found to be −70.1 ± 3.8 mV (Figure 1). This high zeta potential value indicates the stability of the prepared sample, as the charged particles repel one another, preventing their natural tendency to aggregate. Manosroi et al. [40] indicated that negatively charged liposomes exhibited greater stability and a lower leakage rate compared to positively charged liposomes, thereby verifying the stability of the prepared sample. The efficacy of liposomes in biological systems is governed by characteristics such as surface charge, particle size, and lipid composition—factors that collectively influence cellular uptake, stability, and therapeutic properties. Particle size, in particular, dictates the routes of cellular entry; particles ranging from 50 to 200 nm enter via clathrin‐mediated endocytosis, whereas larger particles (~500 nm) preferentially utilize caveolae‐mediated pathways, thereby avoiding lysosomal degradation [41]. The liposomes synthesized in this study, with a hydrodynamic diameter of 289 ± 9.6 nm, fall within a size range suitable for entry into Caco‐2 cells via energy‐dependent uptake. A zeta potential of approximately −70.1 ± 3.8 mV indicates excellent colloidal stability due to strong electrostatic repulsion, which prevents particle aggregation and ensures the long‐term stability of the postbiotic‐loaded liposomes. Although a negative surface charge might theoretically reduce electrostatic adhesion to cell membranes, liposome–cell interaction is not solely dependent on electrostatic forces; it also involves hydrophobic interactions, membrane fluidity, and endocytic pathways. Indeed, as previously mentioned, negatively charged liposomes can still interact with Caco‐2 cells through hydrophobic interactions and lipid raft‐mediated endocytosis [42, 43]. Collectively, these physicochemical properties contribute to the sustained release profile, efficient cellular interaction, and enhanced protective efficacy of our liposomal formulation against gliadin‐induced intestinal damage. Analysis of nanoliposomes encapsulated postbiotic by TEM confirmed the presence of liposomes in a spherical shape with a bilayer membrane structure. According to Gibis et al. [44], the dimensions of the liposomal systems were influenced by the encapsulated bioactive components. This phenomenon can be attributed to the hydrogen bonding interactions between the polar head groups of phospholipids and the components of the bioactive, along with hydrophobic interactions between the lipid fatty acid tails and the hydrophobic regions of the bioactive. Noticeably, the particle size indicated by the TEM image (Figure 1) was smaller than that in DLS analysis because DLS measures time‐dependent fluctuations in scattering intensity generated by particles undergoing Brownian motion, resulting in the mean hydrodynamic diameter.

**Figure 1:**
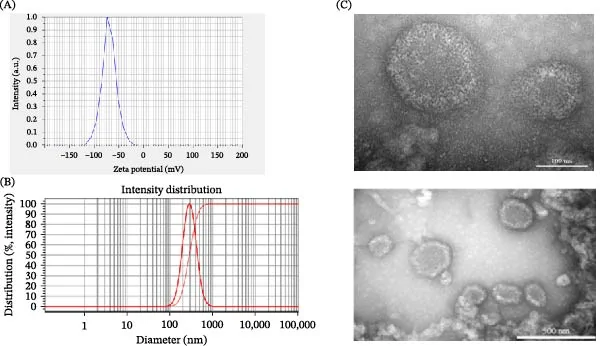
Characterization of liposome‐encapsulated postbiotic. (A) Zeta potential distribution curve showing surface charge of −70.1 ± 3.8 mV. (B) Dynamic light scattering (DLS) histogram showing hydrodynamic diameter (mean: 289 ± 9.6 nm, PDI: 0.13 ± 0.02). (C) Transmission electron microscopy (TEM) image showing spherical morphology and bilayer membrane structure.

### 6.2. EE of Liposome‐Encapsulated Postbiotic

The EE of the postbiotic‐encapsulating liposomes was calculated to be 81.83 ± 3.2%, indicating that ~82% of the postbiotic was successfully incorporated into the nanoliposome structure. Our results are in agreement with those demonstrated by Guan et al. [45], who noted that liposome preparation technique significantly influences the physicochemical properties and loading capacity of active compounds. The EE value obtained in our study is comparable to previous studies using the Mozafari method for encapsulation of bioactive compounds, which reported EE values ranging from 43.51% to 53.71% for green tea polyphenols [46]. A recent study on liposomal encapsulation of probiotics reported EE values of ~86.8% for *L. delbrueckii* [47]. Specifically, in the context of postbiotic encapsulation, a study on liposomal postbiotic formulations reported an optimal EE of 17.52%, indicating that the encapsulation process depends on the nature of the bioactive agent and the formulation conditions [48]. The relatively high EE observed in our study can be attributed to several factors: (a) the aqueous core of the liposomes, which acts as an efficient reservoir for hydrophilic postbiotic metabolites; (b) the stability provided by the liposomal bilayer structure, which prevents leakage of encapsulated components; and (c) the optimized preparation conditions using the Mozafari method, which avoids the use of organic solvents and harsh mechanical forces that could compromise postbiotic integrity [36, 49]. Overall, our results demonstrate that the employed method is an effective approach for the encapsulation of *L. plantarum* postbiotics, achieving EE values that are consistent with or superior to those reported for similar liposomal systems, thereby confirming the suitability of this formulation for further biological evaluations.

### 6.3. Release Profile of the Liposome‐Encapsulated Postbiotic

The drug release profile is commonly assessed to validate formulation quality, provide guidance for dosage regimens, and anticipate the effectiveness of the formulations in vivo. One of the most attractive advantages of liposomal delivery systems is associated with enhanced stability, improved bioavailability, and controlled release of encapsulated bioactives at the desired physiological site. In general, liposomes exhibit biphasic or triphasic release profiles, featuring an initial burst release that is subsequently regulated to improve release characteristics [39]. An in vitro release study was conducted using standard method for evaluating gastrointestinal drug delivery systems and employing PBS at pH levels of 1.2, 6.8, and 7.4 to simulate gastric, intestinal, and systemic conditions, respectively (Figure 2) [50]. The release profile demonstrated a biphasic trend with time and pH dependency. Initially, a burst release was observed (pH 1.2: 55%, pH 6.8: 60%, and pH 7.4: 50%), followed by a slow and gradual release. The initial burst release mainly occurs due to the drug trapped near the surface, while the sustained release results from the diffusion and release of the drug from the liposome core. There was a slight difference in postbiotic release between different pH levels in the first 8 h. Moreover, long‐lasting postbiotic release was observed, indicating the protective role of the liposomal coating on the postbiotic. A rapid release of postbiotic was observed within 24–48 h at the investigated pH levels. The biphasic release pattern identified in our research is anticipated to offer efficient protection for the encapsulated postbiotic during its journey through the gastrointestinal tract while guaranteeing sustained release at the target location; this correlates with the concepts presented by Leghari et al. [51], who highlighted the essential role of lipid‐based delivery systems in enhancing the bioavailability of postbiotics.

**Figure 2:**
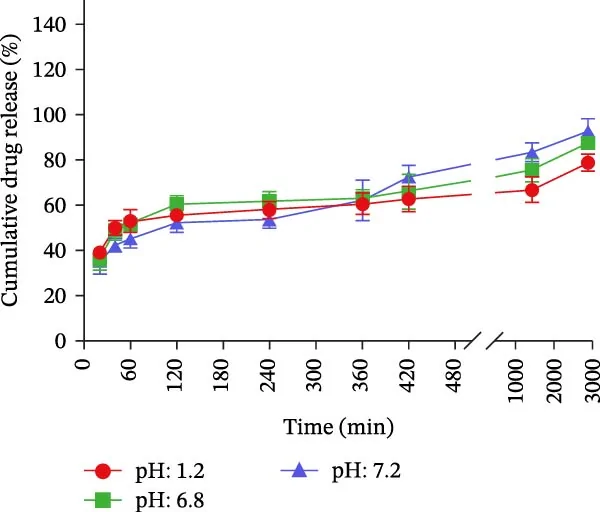
In vitro cumulative release profile of liposome‐encapsulated postbiotic in PBS buffer at different pH values (1.2, 6.8, and 7.4) at 37°C. Data are presented as mean ± SEM (*n* = 3).

### 6.4. Toxicity Evaluation of Free Postbiotic and Liposome‐Encapsulated Postbiotic

The in vitro cytotoxicity of the free postbiotic, liposome‐encapsulated postbiotic, and gliadin was evaluated on epithelial Caco‐2 cells. The Caco‐2 cell line is frequently regarded as a gold standard in vitro model for understanding the role of the intestinal microbiome in the pathogenicity of CD. This cell line is composed of intestinal epithelial cells derived from human colon adenocarcinoma, which, prior to reaching confluence, resembles the physiological enterocytes and serves as a crucial and extensively utilized tool for understanding the molecular and cellular processes involved in the alterations of CD within epithelial cells [52]. The experiment was conducted at two time intervals (24 and 48 h), and Caco‐2 cells were treated with three different concentrations (1, 2, and 4 mg/mL). The cytotoxicity of the free postbiotic, liposome‐encapsulated postbiotic, and gliadin on Caco‐2 cells is shown in Figure 3. Cell viability showed a slight decline as the concentration of both free postbiotic and liposome‐encapsulated postbiotic increased, indicating a dose‐dependent antiproliferative impact on the Caco‐2 cell line. The survival rate of Caco‐2 cells treated with free postbiotic was lower than that of liposome‐encapsulated postbiotic for all applied concentrations. In this study, the observed reduction in cytotoxicity of liposomal postbiotic compared to free postbiotic is due to the slow and prolonged release profile of liposomes, which reduces the initial burst exposure. This important feature is a major advantage in the treatment of CD, where the aim is cell protection rather than cytotoxicity. The findings also showed that free postbiotic and liposome‐encapsulated postbiotic exhibited less toxicity than gliadin in all the investigated concentrations. The MTT assay revealed significant gliadin‐induced cytotoxicity in Caco‐2 cells at 4 mg/mL for 48 h, so that cell viability reduced to 17.8% ±2.11% (*p* = 0.0019) compared with the control group. This study also investigated the effect of employing free and liposomal postbiotics in reducing the toxic effects of gliadin (Figure 4). In this manner, free and liposomal postbiotics at concentrations of 1, 2, and 4 were employed separately in combination with gliadin (4 mg/mL) to treat the cells at two time intervals (24 and 48 h). The results of the MTT assay demonstrated that the cotreatment of free postbiotic with gliadin significantly enhanced cell viability at both time points. Furthermore, cell viability exhibited a positive correlation with increasing concentrations of postbiotic, suggesting a protective role of the postbiotic in reducing the toxic effect of gliadin. The findings from the cotreatment of liposomal postbiotic (1, 2, and 4 mg/mL) with gliadin (4 mg/mL) for 24 h demonstrated outcomes comparable to those observed with free postbiotics in the presence of gliadin. However, a greater protective effect was noted with liposomal postbiotics than with free postbiotics at 48 h. These results indicate that the encapsulation of postbiotics within the liposomal structure enhances their protective efficacy against gliadin toxicity.

**Figure 3:**
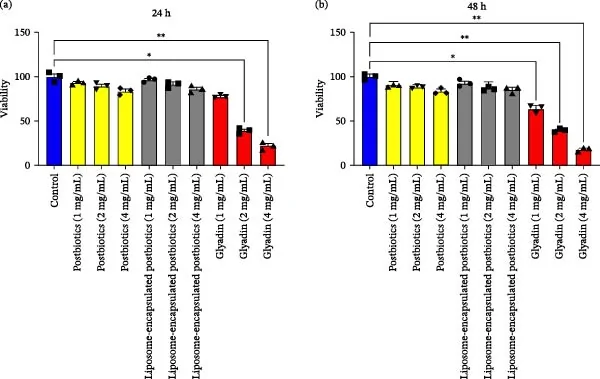
In vitro cytotoxicity of free postbiotic, liposome‐encapsulated postbiotic, and gliadin at different concentrations in Caco‐2 cells determined by the MTT assay. Caco‐2 cells were treated with increasing concentrations of the tested compounds, and cell viability was measured after 24 h (a) and 48 h (b). Data are presented as mean ± SD from three independent experiments. Statistical analysis was performed using the Kruskal–Wallis test followed by Dunn’s post hoc test for multiple comparisons.  ^∗^ *p*  < 0.05 and  ^∗∗^ *p*  < 0.01 indicate significant differences compared with the control group.

**Figure 4:**
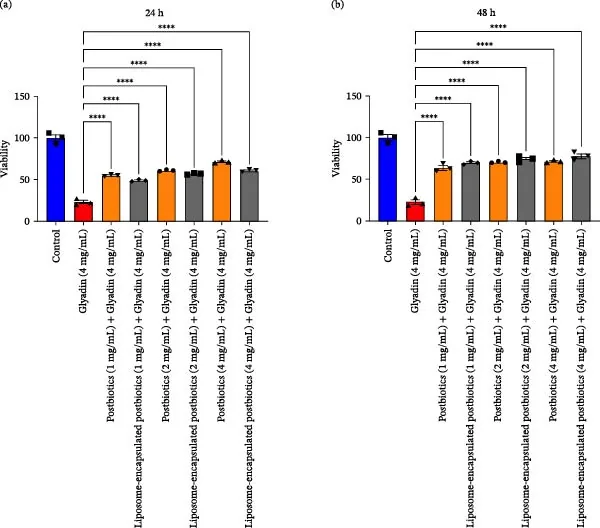
In vitro cytotoxicity of gliadin, cotreatment of free postbiotic with gliadin, and cotreatment of liposome‐encapsulated postbiotic with gliadin in Caco‐2 cells determined by the MTT assay. Cell viability was evaluated after 24 h (a) and 48 h (b) of exposure to the tested treatments. Data are presented as mean ± SD from three independent experiments. Statistical analysis was performed using the Kruskal–Wallis test followed by Dunn’s post hoc test for multiple comparisons.  ^∗∗∗∗^ *p*  < 0.0001 indicates significant differences compared with the control group.

### 6.5. Effects of Gliadin, Free Postbiotic, and Liposome‐Encapsulated Postbiotic Treatments on Zonulin Release

In this study, free postbiotic, liposome‐encapsulated postbiotic, and gliadin were administered to Caco‐2 cells at different times (3, 6, and 12 h). A significant luminal secretion of zonulin was observed after 3 h incubation of Caco‐2 cells with gliadin (1 mg/mL). In the case of postbiotics, the zonulin release was lower than that in Caco‐2 cells treated with gliadin at all investigated time points. The incubation of Caco‐2 cells with liposome‐encapsulated postbiotic led to a significant decrease in zonulin secretion compared to Caco‐2 cells treated with gliadin at three time points. The variations in the zonulin levels were notable between Caco‐2 cells exposed to gliadin and those treated with postbiotic and liposome‐encapsulated postbiotic (Figure 5). The significantly lower zonulin secretion observed with liposomal postbiotic compared to free postbiotic can be assigned to the following factors. First, the slow and sustained release profile (Figure 2) of the liposomal form ensures prolonged exposure of postbiotic components to Caco‐2 cells and maintains their biological activity for a longer period of time. Second, the structure of liposomes facilitates intracellular transport through endocytosis, allowing postbiotic metabolites to reach their intracellular targets more efficiently. Third, the encapsulation process protects sensitive components of postbiotic (e.g., short‐chain fatty acids and peptides) from environmental damage. Taken together, all these mechanisms improve the ability of liposomal postbiotic to inhibit the zonulin pathway and maintain TJ integrity. Orlando et al. [52] have observed that the cotreatment of viable *Lactobacillus rhamnosus* GG (L.GG), heat‐killed L.GG (L.GG‐HK), or its conditioned medium (L.GG‐CM) with gliadin remarkably improved barrier function as evidenced by lactulose flux, transepithelial resistance, and zonulin release. The findings of this research indicate the encouraging potential of postbiotics in lowering zonulin release and consequently alleviating symptoms of CD.

**Figure 5:**
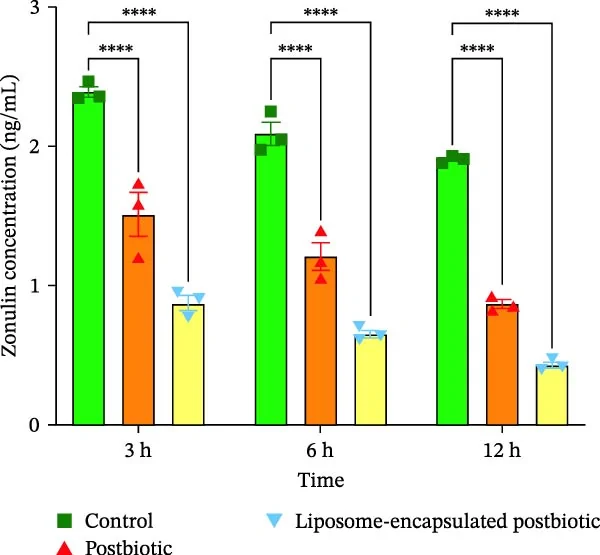
Zonulin release in Caco‐2 monolayers exposed to gliadin (1 mg/mL) alone or in combination with free postbiotic and liposome‐encapsulated postbiotic. Zonulin concentration was measured after 3, 6, and 12 h of treatment. Data are presented as mean ± SD from three independent experiments. Statistical analysis was performed using the Kruskal–Wallis test followed by Dunn’s post hoc test for multiple comparisons.  ^∗∗∗∗^ *p*  < 0.0001 indicates significant differences compared with the control group.

## 7. Conclusion

Several studies have indicated the crucial importance of probiotics in preventing the harmful effects of gliadin and gliadin peptides. However, fewer studies have assessed the role of postbiotics in CD. Given the benefits highlighted for postbiotics, we used the postbiotic from a widely known probiotic strain, *L. plantarum*, in managing CD. The liposomal formulation reported here demonstrates high EE and sustained release profile, which makes it an efficient drug delivery system. The findings of this research exhibited a dose‐dependent cytotoxicity of postbiotic, liposome‐encapsulated postbiotic, and gliadin towards Caco‐2 cells. In addition, MTT assay demonstrated that gliadin exhibited significant cytotoxicity against Caco‐2 cells, while postbiotic and its liposomal form exhibited negligible toxicity, and the cell survival was nearly 90%. The ELISA indicated that there were significant differences in zonulin levels between cells exposed to gliadin and those treated with free postbiotic and liposome‐encapsulated postbiotic. In conclusion, the liposome‐encapsulated postbiotic from *L. plantarum* significantly contributes to safeguarding the intestinal mucosa from damage caused by gliadin, making it a promising candidate for CD treatment. Nevertheless, additional research is necessary to elucidate the components of the mentioned postbiotics, and emphasizing their role in alleviating CD is highly recommended. Finally, in vivo studies in animal models could confirm the efficacy of the obtained results.

## Author Contributions

**Mojdeh Safari:** conceptualization, writing – original draft, visualization. **Mahdi Adabi**: conceptualization, writing – review and editing. **Naheed Mojgani**: conceptualization, methodology, validation. **Fatemeh Pirnajafi and Mohaddeseh Khadem**: methodology and investigation. **Milad Sadeghzadeh**: formal analysis and software. **Masoomeh Amini and Bijan Shahbazkhani**: project administration, supervision, resources.

## Funding

This research was funded by the corresponding authors.

## Conflicts of Interest

The authors declare no conflicts of interest.

## Data Availability

Data will be made available upon request.

## References

[1] Sollid L. M. , Coeliac Disease: Dissecting a Complex Inflammatory Disorder, Nature Reviews Immunology. (2002) 2, no. 9, 647–655. 10.1038/nri885 12209133

[2] Kagnoff M. F. , Celiac Disease: Pathogenesis of a Model Immunogenetic Disease, The Journal of Clinical Investigation. (2007) 117, no. 1, 41–49. 10.1172/JCI30253 PMC1716218 17200705

[3] Sallese M. , Lopetuso L. R. , Efthymakis K. , and Neri M. , Beyond the Hla Genes in Gluten-Related Disorders, Frontiers in Nutrition. (2020) 7, 575844. 10.3389/fnut.2020.575844 PMC7688450 33262997

[4] Gujral N. , Freeman H. J. , and Thomson A. B. R. , Celiac Disease: Prevalence, Diagnosis, Pathogenesis and Treatment, World Journal of Gastroenterology. (2012) 18, no. 42, 6036–6059. 10.3748/wjg.v18.i42.6036 PMC3496881 23155333

[5] Lerner A. , New Therapeutic Strategies for Celiac Disease, Autoimmunity Reviews. (2010) 9, no. 3, 144–147. 10.1016/j.autrev.2009.05.002 19427921

[6] Fasano A. , Zonulin and Its Regulation of Intestinal Barrier Function: The Biological Door to Inflammation, Autoimmunity, and Cancer, Physiological Reviews. (2011) 91, no. 1, 151–175. 10.1152/physrev.00003.2008 21248165

[7] Wood Heickman L. K. , DeBoer M. D. , and Fasano A. , Zonulin as a Potential Putative Biomarker of Risk for Shared Type 1 Diabetes and Celiac Disease Autoimmunity, Diabetes/Metabolism Research and Reviews. (2020) 36, no. 5, e3309. 10.1002/dmrr.3309 PMC7340576 32162764

[8] Tripathi A. , Lammers K. M. , and Goldblum S. , et al.Identification of Human Zonulin, a Physiological Modulator of Tight Junctions, as Prehaptoglobin-2, Proceedings of the National Academy of Sciences of the United States of America. (2009) 106, no. 39, 16799–16804. 10.1073/pnas.0906773106 PMC2744629 19805376

[9] Barbaro M. R. , Cremon C. , and Morselli-Labate A. M. , et al.Serum Zonulin and Its Diagnostic Performance in Non-Coeliac Gluten Sensitivity, Gut. (2020) 69, no. 11, 1966–1974. 10.1136/gutjnl-2019-319281 32060130

[10] Drago S. , El Asmar R. , and Di Pierro M. , et al.Gliadin Zonulin and Gut Permeability: Effects on Celiac and Non-Celiac Intestinal Mucosa and Intestinal Cell Lines, Scandinavian Journal of Gastroenterology. (2006) 41, no. 4, 408–419. 10.1080/00365520500235334 16635908

[11] Caio G. , Ciccocioppo R. , Zoli G. , De Giorgio R. , and Volta U. , Therapeutic Options for Coeliac Disease: What Else Beyond Gluten-Free Diet?, Digestive and Liver Disease. (2020) 52, no. 2, 130–137. 10.1016/j.dld.2019.11.010 31831308

[12] Mäki M. , Celiac Disease Treatment: Gluten-Free Diet and Beyond, Journal of Pediatric Gastroenterology and Nutrition. (2014) 59, S15–S17. 10.1097/01.mpg.0000450397.76521.f9 24979194

[13] Schuppan D. , Junker Y. , and Barisani D. , Celiac Disease: From Pathogenesis to Novel Therapies, Gastroenterology. (2009) 137, no. 6, 1912–1933. 10.1053/j.gastro.2009.09.008 19766641

[14] Conte M. , Porpora M. , and Nigro F. , et al.Pro-Pre and Postbiotic in Celiac Disease, Applied Sciences. (2021) 11, no. 17, 8185.

[15] da Silva T. F. , de Assis Glória R. , and Americo M. F. , et al.Unlocking the Potential of Probiotics: A Comprehensive Review on Research, Production, and Regulation of Probiotics, Probiotics and Antimicrobial Proteins. (2024) 16, no. 5, 1687–1723. 10.1007/s12602-024-10247-x 38539008

[16] Jenickova E. , Andrén Aronsson C. , Mascellani Bergo A. , Cinek O. , Havlik J. , and Agardh D. , Effects of Lactiplantibacillus Plantarum and Lacticaseibacillus Paracasei Supplementation on the Faecal Metabolome in Children With Coeliac Disease Autoimmunity: A Randomised, Double-Blinded Placebo-Controlled Clinical Trial, Frontiers in Nutrition. (2023) 10, 1183963. 10.3389/fnut.2023.1183963 PMC10359497 37485388

[17] Doguer C. , Demirok N. T. , and Gunduz K. B. E. , Restorative Effects of Acetobacter Ghanensis on the Pathogenicity of Gliadin-Induced Modulation of Tight Junction-Associated Gene Expression in Intestinal Epithelial Cells: Impacts of Acetobacter Ghanensis on the Intestinal Epithelial Barrier, Journal of Surgery and Medicine. (2023) 7, no. 2, 133–137.

[18] Håkansson Å. , Andrén Aronsson C. , Brundin C. , Oscarsson E. , Molin G. , and Agardh D. , Effects of *Lactobacillus plantarum* and *Lactobacillus paracasei* on the Peripheral Immune Response in Children With Celiac Disease Autoimmunity: A Randomized, Double-Blind, Placebo-Controlled Clinical Trial, Nutrients. (2019) 11, no. 8, 1925. 10.3390/nu11081925 PMC6723580 31426299

[19] Dargenio V. N. , Castellaneta S. , and Panico S. , et al.Probiotics and Gastrointestinal Diseases, Minerva Pediatrics. (2022) 74, no. 6, 703–723, 10.23736/S2724-5276.22.07031-8. 36315413

[20] Thantsha M. S. , Mamvura C. I. , and Booyens J. , Probiotics—What They Are, Their Benefits and Challenges, New Advances in the Basic and Clinical Gastroenterology, 2012, 10.5772/32889.

[21] Terpou A. , Papadaki A. , Lappa I. K. , Kachrimanidou V. , Bosnea L. A. , and Kopsahelis N. , Probiotics in Food Systems: Significance and Emerging Strategies Towards Improved Viability and Delivery of Enhanced Beneficial Value, Nutrients. (2019) 11, no. 7, 1591. 10.3390/nu11071591 PMC6683253 31337060

[22] Salminen S. , Collado M. C. , and Endo A. , et al.The International Scientific Association of Probiotics and Prebiotics (ISAPP) Consensus Statement on the Definition and Scope of Postbiotics, Nature Reviews Gastroenterology & Hepatology. (2021) 18, no. 9, 649–667. 10.1038/s41575-021-00440-6 PMC8387231 33948025

[23] Khani N. , Bonyadi M. , Soleimani R. A. , Raziabad R. H. , Ahmadi M. , and Homayouni-Rad A. , et al.Postbiotics: as a Promising Tools in the Treatment of Celiac Disease, Probiotics and Antimicrobial Proteins. (2025) 17, no. 3, 1513–1522. 10.1007/s12602-024-10416-y 39673575

[24] Sarno M. , Lania G. , and Cuomo M. , et al. *Lactobacillus paracasei* CBA L74 Interferes With Gliadin Peptides Entrance in Caco-2 Cells, International Journal of Food Sciences and Nutrition. (2014) 65, no. 8, 953–959. 10.3109/09637486.2014.940283 25030417

[25] Balendra V. , Rosenfeld R. , and Amoroso C. , et al.Postbiotics as Adjuvant Therapy in Cancer Care, Nutrients. (2024) 16, no. 15, 10.3390/nu16152400, 2400. PMC11314502 39125280

[26] Rajam R. and Subramanian P. , Encapsulation of Probiotics: Past, Present and Future, Beni-Suef University Journal of Basic and Applied Sciences. (2022) 11, no. 1, 46.

[27] Reque P. M. and Brandelli A. , Encapsulation of Probiotics and Nutraceuticals: Applications in Functional Food Industry, Trends in Food Science & Technology. (2021) 114, 1–10.

[28] Agriopoulou S. , Tarapoulouzi M. , Varzakas T. , and Jafari S. M. , Application of Encapsulation Strategies for Probiotics: From Individual Loading to Co-Encapsulation, Microorganisms. (2023) 11, no. 12, 2896. 10.3390/microorganisms11122896 PMC10745938 38138040

[29] Sun Q. , Yin S. , He Y. , Cao Y. , and Jiang C. , Biomaterials and Encapsulation Techniques for Probiotics: Current Status and Future Prospects in Biomedical Applications, Nanomaterials. (2023) 13, no. 15, 2185. 10.3390/nano13152185 PMC10421492 37570503

[30] Han M. , Yang S. , Song J. , and Gao Z. , Layer-by-Layer Coated Probiotics With Chitosan and Liposomes Demonstrate Improved Stability and Antioxidant Properties In Vitro, International Journal of Biological Macromolecules. (2024) 258, 128826. 10.1016/j.ijbiomac.2023.128826 38123040

[31] Guimarães D. , Cavaco-Paulo A. , and Nogueira E. , Design of Liposomes as Drug Delivery System for Therapeutic Applications, International Journal of Pharmaceutics. (2021) 601, 120571. 10.1016/j.ijpharm.2021.120571 33812967

[32] Lu W.-L. and Qi X.-R. , Liposome-Based Drug Delivery Systems, 2021, Springer.

[33] Jalali S. , Mojgani N. , Haghighat S. , Sanjabi M. R. , and Sarem-Nezhad S. , Investigation of Antimicrobial and Antioxidant Properties of Postbiotics Produced by *Lactobacillus rhamnosus* and Limosilactobacillus Reuteri and Their Potential Application in Surface Decontamination of Red Meat, LWT - Food Science and Technology. (2024) 209, 116758.

[34] Moradi M. , Molaei R. , and Guimarães J. T. , A Review on Preparation and Chemical Analysis of Postbiotics From Lactic Acid Bacteria, Enzyme and Microbial Technology. (2021) 143, 109722. 10.1016/j.enzmictec.2020.109722 33375981

[35] Rasti B. , Jinap S. , Mozafari M. R. , and Yazid A. M. , Comparative Study of the Oxidative and Physical Stability of Liposomal and Nanoliposomal Polyunsaturated Fatty Acids Prepared With Conventional and Mozafari Methods, Food Chemistry. (2012) 135, no. 4, 2761–2770. 10.1016/j.foodchem.2012.07.016 22980870

[36] Mozafari M. R. , Weissig V. , Nanoliposomes: Preparation and Analysis, Liposomes: Methods and Protocols, Volume 1: Pharmaceutical Nanocarriers, 2010, Humana Press, 29–50, 10.1007/978-1-60327-360-2_2.

[37] Noble J. E. and Bailey M. J. , Quantitation of Protein, Methods in Enzymology. (2009) 463, 73–95. 10.1016/S0076-6879(09)63008-1 19892168

[38] Chang H. M. , Foo H. L. , Loh T. C. , Lim E. T. C. , and Abdul Mutalib N. E. , Comparative Studies of Inhibitory and Antioxidant Activities, and Organic Acids Compositions of Postbiotics Produced by Probiotic Lactiplantibacillus Plantarum Strains Isolated From Malaysian Foods, Frontiers in Veterinary Science. (2021) 7, 602280. 10.3389/fvets.2020.602280 PMC7870707 33575277

[39] Zarrabi A. , Zarepour A. , Khosravi A. , Alimohammadi Z. , and Thakur V. K. , Synthesis of Curcumin Loaded Smart Ph-Responsive Stealth Liposome as a Novel Nanocarrier for Cancer Treatment, Fibers. (2021) 9, no. 3, 19.

[40] Manosroi A. , Kongkaneramit L. , and Manosroi J. , Stability and Transdermal Absorption of Topical Amphotericin B Liposome Formulations, International Journal of Pharmaceutics. (2004) 270, 79–286. 10.1016/j.ijpharm.2003.10.031 14726142

[41] Rejman J. , Oberle V. , Zuhorn I. S. , and Hoekstra D. , Size-Dependent Internalization of Particles via the Pathways of Clathrin-and Caveolae-Mediated Endocytosis, The Biochemical Journal. (2004) 377, no. 1, 159–169. 10.1042/BJ20031253 PMC1223843 14505488

[42] Bozzuto G. and Molinari A. , Liposomes as Nanomedical Devices, International Journal of Nanomedicine. (2015) 10, 975–999. 10.2147/IJN.S68861 PMC4324542 25678787

[43] Ngo K. X. , Umakoshi H. , Shimanouchi T. , and Kuboi R. , Characterization of Heat-Induced Interaction of Neutral Liposome With Lipid Membrane of *Streptomyces griseus* Cell, Colloids and Surfaces B: Biointerfaces. (2009) 73, no. 2, 399–407. 10.1016/j.colsurfb.2009.06.015 19592227

[44] Gibis M. , Vogt E. , and Weiss J. , Encapsulation of Polyphenolic Grape Seed Extract in Polymer-Coated Liposomes, Food & Function. (2012) 3, no. 3, 246–254. 10.1039/c1fo10181a 22116575

[45] Guan Y. , Zhu H. , Lin M. , Zhang Y. , He Q. , and Pan J. , Preparation and Stability Evaluation of Flexible Nanoliposomes Co-Encapsulated With Black Wolfberry Anthocyanins and EGCG, LWT - Food Science and Technology. (2025) 217, 117402.

[46] Jahanfar S. H. , Gahavami M. , Khosravi-Darani K. , and Jahadi M. , Liposomal Green Tea Extract: Optimization and Physicochemical Characterization, Journal of Applied Biotechnology Reports. (2020) 10.30491/JABR.2020.231423.1228.

[47] Gökçe H. B. and Aslan İ. , Novel Liposome–Gel Formulations Containing a Next Generation Postbiotic: Characterization, Rheological, Stability, Release Kinetic, and In Vitro Antimicrobial Activity Studies, Gels. (2024) 10, no. 11, 746. 10.3390/gels10110746 PMC11593572 39590102

[48] Liu J. , Sun Z. , and Liu S. , et al.Liposome-Mediated Encapsulation of Probiotics: Current Status, Challenges and Future Directions, Critical Reviews in Food Science and Nutrition. (2025) 65, no. 31, 7792–7809. 10.1080/10408398.2025.2480169 40176707

[49] Ghorbanzade T. , Jafari S. M. , Akhavan S. , and Hadavi R. , Nano-Encapsulation of Fish Oil in Nano-Liposomes and Its Application in Fortification of Yogurt, Food Chemistry. (2017) 216, 146–152. 10.1016/j.foodchem.2016.08.022 27596403

[50] Zhang Y. , Wang Y. , and Lu Y. , et al.Advanced Oral Drug Delivery Systems for Gastrointestinal Targeted Delivery: The Design Principles and Foundations, Journal of Nanobiotechnology. (2025) 23, 400. 10.1186/s12951-025-03479-8 PMC12124048 40448152

[51] Leghari A. , Khand F. M. , Laghari S. , and Lakho S. A. , Engineering Bioavailability: Polysaccharide-Protein-Lipid Delivery Platforms for Postbiotic Therapeutics in Gastrointestinal and Systemic Disorders, International Journal of Biological Macromolecules. (2026) 351, 151123. 10.1016/j.ijbiomac.2026.151123 41759844

[52] Orlando A. , Linsalata M. , Notarnicola M. , Tutino V. , and Russo F. , Lactobacillus GG Restoration of the Gliadin Induced Epithelial Barrier Disruption: The Role of Cellular Polyamines, BMC Microbiology. (2014) 14, no. 1, 19. 10.1186/1471-2180-14-19 PMC3911798 24483336

